# The parasitoids of *Parlatoria ziziphi* (Lucas) (Hemiptera: Diaspididae): with descriptions of two new species of *Aphytis* (Hymenoptera: Aphelinidae) from China, and a note on *Aphytis* species with black-tipped mid tibiae in the male

**DOI:** 10.3390/insects16101070

**Published:** 2025-10-20

**Authors:** Jingtao Xi, Junqing Ge, Jian Huang, Andrew Polaszek, Zhuhong Wang

**Affiliations:** 1 College of Plant Protection, Fujian Agriculture and Forestry University, Fuzhou 350002, China; 12302003026@fafu.edu.cn (J.X.); jhuang1234@126.com (J.H.); 2 Institute of Biotechnology, Fujian Academy of Agricultural Sciences, Fuzhou 350003, China; jqge@163.com; 3 Science: Research, Natural History Museum, London SW7 5BD, UK; a.polaszek@nhm.ac.uk

**Keywords:** black parlatoria scale, parasitoids, biocontrol, 28SrDNA sequences, taxonomy

## Abstract

During investigations in the Fujian and Hunan Provinces of China, 5 parasitoids associated with *Parlatoria ziziphi* were identified based on morphological characteristics and 28SrDNA sequences. They comprised 4 ectoparasitoids: *A. chrysomphali* (Mercet), *Aphytis jinshanensis* Wang & Huang, **sp.n.**, *Aphytis nigromaculata* Wang & Huang, **sp.n.**, and *A.* nr. *melinus* DeBach, and 1 endoparasitoid, *Encarsia citrina* (Craw). The 2 new species are described and illustrated in detail, and the relationships of the 4 *Aphytis* parasitoids treated here are analyzed, resulting in a phylogenetic tree based on 28SrDNA sequences. Moreover, the 5 *Aphytis* species (including 1 new species described in this work) having the mid-tibia tipped with black in the male are summarized with their scale hosts and distribution, and an identification key to these species is provided.

## Simple Summary

The black parlatoria scale, *Parlatoria ziziphi* (Lucas) (Hemiptera: Diaspididae), is a destructive pest attacking citrus plants worldwide. Parasitoids are important natural enemies used for the biological control of *P. ziziphi*. In Egypt from 2005 to 2007, the parasitoid, *Aphytis melinus* DeBach, was mass reared and released in large numbers to control *P. ziziphi*. Currently, 10 parasitoids associated with *P. ziziphi* have been recorded worldwide, including 8 species of Aphelinidae (including 1 hyperparasitoid) and 2 species of Encyrtidae. In China, 3 parasitoids are recorded, 2 Aphelinidae and 1 Encyrtidae. This study investigates and identifies 5 parasitoids in Fujian and Hunan Provinces of China, including 2 new species of *Aphytis*. Information on the 5 *Aphytis* species having the mid-tibia tipped with black in the male is summarized, and an identification key to these species is provided.

## 1. Introduction

The black parlatoria scale, *Parlatoria ziziphi* (Lucas) (Hemiptera: Diaspididae), is a common pest in tropical and subtropical regions, occurring mainly in citrus growing areas of East and Southeast Asia, the Middle East, South America, Africa, and the Mediterranean coast [1,2,3]. In Tunisia, *P. ziziphi* was one of the four major economic pests, posing a severe threat to the local citrus industry [4]. In Egypt, *P. ziziphi* has been the most serious pest on citrus since the 1990s [5]. According to reports, *P. ziziphi* caused heavy economic losses in some citrus growing countries such as Algeria, Morocco and France [6,7], and was also listed as a key quarantine target in the international citrus trade by some countries and regional organizations [1]. In China, *P. ziziphi* damages citrus plants, developing 3–4 generations per year and overlapping generations, seriously impacting citrus yields [8,9].

Parasitoids are important natural enemies for biological control of *Parlatoria ziziphi*. Currently, a total of 10 species of parasitoids are known associated with *P. ziziphi* all over the world, including 8 species of Aphelinidae (including 1 hyperparasitoid) and 2 species of Encyrtidae (Table 1). In Egypt, *Encarsia citrina* (Craw) was considered to be a biocontrol agent with high potential [10], and *Aphytis melinus* DeBach was mass reared and released in many regions of Egypt from 2005 to 2007, successfully colonizing and controlling *P. ziziphi* [11].

Three parasitoids of *Parlatoria ziziphi* are currently recorded from China, 2 Aphelinidae and 1 Encyrtidae (Table 1). The parasitoid behaviour and annual occurrence dynamics of *Encarsia citrina* were studied by Lei et al. (1988) [15] and Ren et al. (1988) [16]. In this paper, 5 parasitoids of *P. ziziphi* are investigated and identified from citrus plants in Fujian and Hunan Provinces. Two new species, *Aphytis jinshanensis* Wang & Huang, **sp.n.** and *Aphytis nigromaculata* Wang & Huang, **sp.n.** are described and illustrated. A phylogenetic tree based on 28SrDNA sequences is constructed to analyze the relationships of the 4 *Aphytis* parasitoids treated in this work. Moreover, the 5 *Aphytis* species (including 1 new species described here) having the mid-tibia tipped with black in the male are summarized with their scale hosts and distribution (Table 2), and an identification key to species is provided.

## 2. Materials and Methods

### 2.1. Collection of Parasitoids

*Parlatoria ziziphi* specimens were collected on citrus plants in Fujian and Hunan Provinces, and observed in the laboratory for parasitoid emergence. The parasitoids reared from these scales were preserved in 100% ethanol after emergence. For *Aphytis* parasitoids, the full-grown larvae or pupae were removed from the parasitized scale hosts before emergence, and placed each in an individually numbered plastic box (3 cm in diameter, 1.5 cm high), in which a filter paper was placed and a drop of water added daily to maintain humidity. The pigmentation of mature pupae, with green eyes, was then observed and photographed, and later used as an important supplementary diagnostic character for identification of *Aphytis* species.

### 2.2. Photographs and Slides of Parasitoids

Body colour of specimens, and the appearance of the propodeal crenulae and thoracic tergum of *Aphytis* specimens were described and photographed before slide-mounting. Specimens were slide-mounted for species identification following the method outlined by Noyes (1982) [23]. Specimens were photographed using a Nikon DS-Ri2 camera (Tokyo, Japan), with NIS-Elements Dv4.40 software, attached to a Nikon SMZ18 microscope. Slide-mounted specimens were photographed by a Nikon Ni microscope with the same camera and software. Type material and the other specimens examined in the study are deposited in the College of Plant Protection, Fujian Agriculture and Forestry University, Fuzhou, Fujian, China (FAFU).

### 2.3. DNA Sequencing and Phylogenetic Analysis

Genomic DNA was extracted using a non-destructive protocol following the method of Polaszek et al. (2013) [24]. Using a DNeasy^®^ blood and tissue kit (Qiagen, Hilden, Germany), the extracted specimens were subsequently used for slide-mounting. The 28SrDNA-D2 expansion region was amplified with PCR using the D2-3551F/D2-4068R primer pair of Wang et al. (2024) [25]. Primer sequences and cycling conditions are given in Table 3. Reactions were performed in 25 µL volumes containing 12.5 µL 2× Taq PCR MasterMix II, 1 µL forward primer, 1 µL reverse primer, 2 µL genomic DNA extract and 8.5 µL ddH_2_O. All amplifications were confirmed by 1% agarose gel electrophoresis. DNA was sequenced at Sangon Biotech (Shanghai, China) using the same primers as for the PCR. Sequences were edited and verified using DNAMAN version 9.0 comparing forward and reverse sequences. All sequences were submitted to GenBank (Table 4).

Relevant reference sequences were downloaded from Genbank/NCBI, and all sequences used for analysis are shown in Table 4. The corresponding sequences were compared using MAFFT v7.505 [26], the aligned sequences were used to construct Bayesian phylogenetic tree with the MrBayes v3.2.7a [27]. Analyses were run using a GTR + F + I + G4 model of nucleotide substitution as this was determined as the most appropriate model with ModelFinder v2.2.0 [28]. All phylogenetic analyses of sequences were conducted on Phylosiuit v1.2.3 software [29].

### 2.4. Terminology and Abbreviations

Terminology follows Rosen and DeBach (1979) [22] with some modification, and the following abbreviations are used: F1, F2, etc. = funicle antennomeres 1, 2, etc.; T1, T2, etc. = gastral tergites 1, 2, etc.

Abbreviations for depositories of specimens are as follows:
FAFUCollege of Plant Protection, Fujian Agriculture and Forestry University, Fuzhou, Fujian, ChinaMNCNInstituto Espanol de Entomologia, Museo Nacional de Ciencias Naturales, Madrid, SpainUSNMUnited States National Museum of Natural History, Washington, DC, USA

## 3. Results

### 3.1. Parlatoria ziziphi and Pupae or Mature Larvae of Parasitoids (Figure 1 and Figure 2)

**Figure 1:**
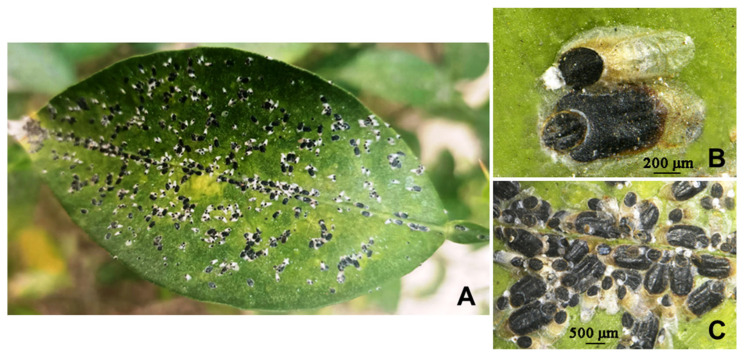
*Parlatoria ziziphi*. (**A**) scales densely covering the leaf; (**B**) above, male scale; below, female scale; (**C**) scale population.

**Figure 2:**
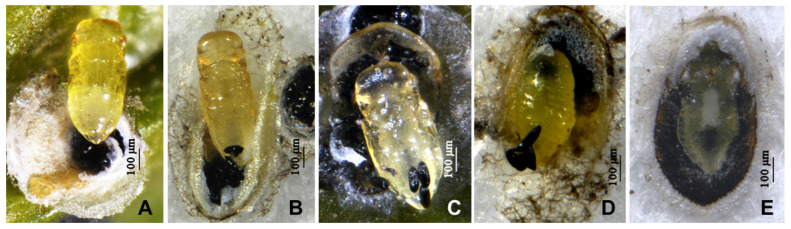
The pupae or mature larvae of parasitiods. (**A**) *Aphytis nigromaculata*, **sp.n.**, pupa; (**B**) *A. jinshanensis*, **sp.n.**, pupa; (**C**) *A. chrysomphali*, pupa; (**D**) *A.* nr. *melinus*, mature larva; (**E**) *Encarsia citrina*, mature larva.

*Parlatoria ziziphi* causes damage to citrus and pomelo plants by densely assembling on the leaves (Figure 1), leading to leaf drop and dead branch in severe cases. Female scales cover the hard shells, while male mostly soft. So the parasitoids primarily parasitize the male scales, although *Encarsia citrina* can be observed emerging from the male and female scales. The pupae or mature larvae of the 5 parasitoids on *P. ziziphi* are shown in Figure 2. The 4 species of *Aphytis* are ectoparasitoids, and *E. citrina* is an endoparasitoid.

### 3.2. Species Accounts

#### 3.2.1. *Aphytis nigromaculata* Wang & Huang, **sp.n.** (Figure 3, Figure 4, Figure 5 and Figure 6)

Zoobank:urn:lsid:zoobank.org:act:02D93CE7-CB93-4355-8B37-2F81B6E2BD6F

*Diagnosis. Aphytis nigromaculata*, **sp.n.** belongs to the *chrysomphali* group and resembles *A. mazalae* DeBach & Rosen in having the tip of middle tibia black in the male, but can be distinguished from it mainly by male characters and pupal pigmentation: *A. nigromaculata*, **sp.n.**: antenna with F3 in the male obliquely truncate from both the dorsal and ventral to the central, forming a point (*A. mazalae*: F3 obliquely truncate from the dorsal to the ventral, ventral aspect considerably longer than the dorsal); *A. nigromaculata*, **sp.n.**: pupa characterized by brown to dark brown on the head and thorax, the male pupa darker (*A. mazalae*: pupa entirely yellow).

*Description*. **Female.** Body length 0.70–0.76 mm. **Colour.** Body yellow, mandibles black-brown; posterior margin of scutellum narrowly lined with black; thoracic sterna yellow. Leg and antenna concolorous with body, antennal pedicel, funicle and club faintly dusky, club dusky at apex, the tarsi of legs faintly dusky. Fore wing hyaline. **Head.** Eyes finely setose. Mandible with three sharp denticles. Antennal formula 1,1,3,1; scape slender, 4.75–6.00× as long as wide, 1.15× as long as club; pedicel 1.79–1.86× as long as wide, longer than F3; F1 and F2 small; F3 rectangular, 0.93–1.12× as long as wide, bearing 1 longitudinal sensillum; club 1.97–2.52× as long as wide, 2.28–2.50× as long as F3, bearing 4 longitudinal sensilla. **Mesosoma.** Mid lobe of mesoscutum with 10 setae; each side lobe of mesoscutum with 2 setae; each axilla with 1 seta; scutellum with 4 setae, the placoid sensilla usually closer to the anterior pair setae, scutellum 0.76–0.83× as long as the mid lobe of mesoscutum; Propodeum long, 0.79–0.89× as long as the scutellum, reticulate broadly on the central area, posterior margin slightly protruding, crenulae small and round, 4 + 4 to 7 + 5 forming 2 sets, nonoverlapping. **Fore wing.** 2.50–2.79× as long as wide; marginal fringe not exceeding 0.33× width of disk; delta area with 35 setae in 5 rows; submarginal vein bearing 2 setae; marginal vein bearing 8–10 setae along anterior margin. **Leg.** Mid-tibial spur nearly as long as the corresponding basitarsus; tarsal formula 5-5-5. **Metasoma.** Petiole and T1–T5 reticulate laterally, with transverse striation centrally. T1–T6 bearing 2 setae on each side, T5 and T6 with 2 setae centrally, T6 mostly reticulate, T7 triangular, indistinctly reticulate-striated, bearing 7 or 8 setae in 1 row. Ovipositor located basally at T3, 1.60–1.67× as long as mid tibia, third valvula short, 0.45–0.53× as long as mid-tibia.

**Male**. Body length 0.66–0.69 mm. Essentially similar to the female in structure, chaetotaxy, sculpture and colour, differing mainly in the colour of the middle tibia and the structure of the antenna. Antennal pedicel 1.55× as long as wide; F1 and F2 small; F3 obliquely truncate from both the dorsal and ventral to the central, forming a point, 1.65× as long as wide, bearing 1 longitudinal sensillum; club 2.19× as long as wide, 1.39× as long as F3, bearing 2 longitudinal sensilla. Mide lobe of mesoscutum with 10 setae, each side lobe with 2 setae; each axilla with 1 seta; scutellum with 4 setae, the placoid sensilla close to the posterior pair of setae. Propodeum 0.67× as long as the scutellum, crenulae as in the female. Fore wing 2.95× as long as wide, marginal fringe not exceeding 0.31× width of disk; delta area with 35 setae in 5 rows; submarginal vein bearing 2 setae; marginal vein bearing 10 setae along anterior margin. Male genitalia 0.67× as long as mid-tibia.

**Pupa.** The pupa of *A. nigromaculata*, **sp.n.** is characterized by brown to dark brown head and thorax; the gaster mostly yellow. The colour of the male pupa is darker, with the black tip of the middle tibia.

*Host. Parlatoria ziziphi* (Lucas) on citrus.

*Distribution*. China (Fujian).

*Etymology*. The species name is derived from the Latin, *nigro* = black, *maculata* = patched, referring to a black patch on the tip of the middle tibia in the male.

Material. **Holotype** ♀, China: Fujian, Fuzhou, Jinshan, FAFU, 9.iii.2018, **ex.** *Parlatoria ziziphi* (Lucas) on citrus. coll. Yu Si and Junhui Zhou (FAFU); **Paratypes** 4♀2♂, same data as holotype (FAFU).

Note. *A. nigromaculata*, **sp.n.** parasitizes the male of *P. ziziphi*.

**Figure 3:**
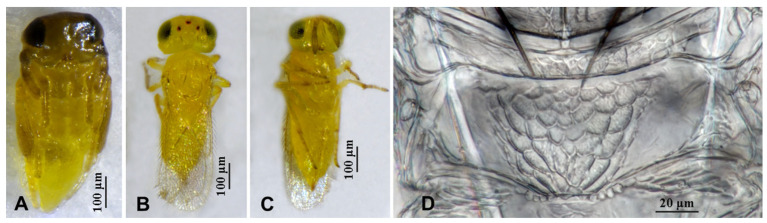
*Aphytis nigromaculata*, **sp.n.**, female. (**A**) pupa in ventral view; (**B**) adult in dorsal view; (**C**) adult in ventral view; (**D**) metanotum and propodeum.

**Figure 4:**
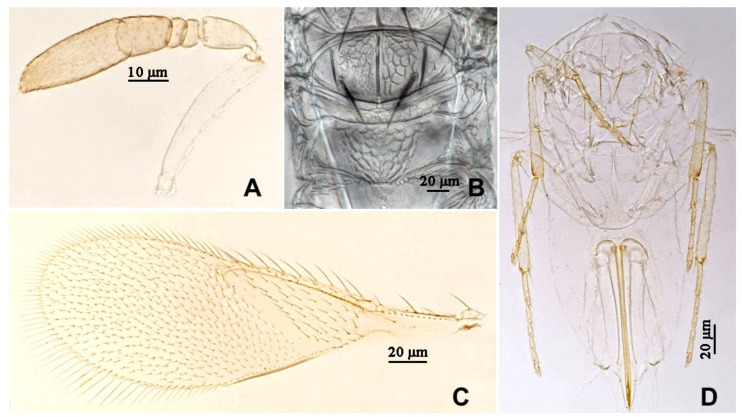
*Aphytis nigromaculata*, **sp.n.**, female. (**A**) antenna; (**B**) mesonotum and propodeum; (**C**) fore wing; (**D**) mesosoma and metasoma in ventral view.

**Figure 5:**
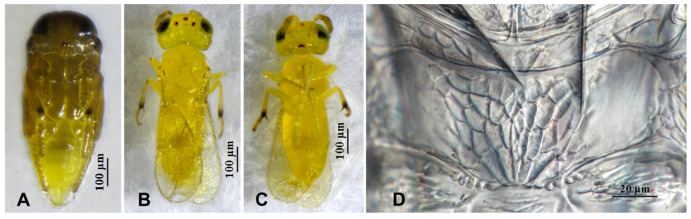
*Aphytis nigromaculata*, **sp.n.**, male. (**A**) pupa in ventral view; (**B**) adult in dorsal view; (**C**) adult in ventral view; (**D**) metanotum and propodeum.

**Figure 6:**
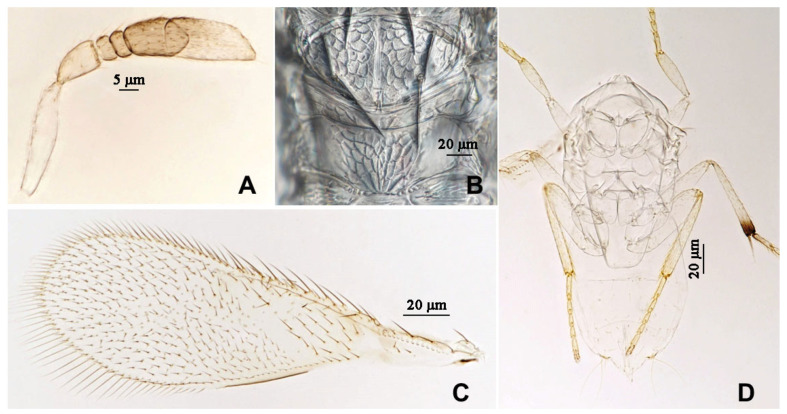
*Aphytis nigromaculata*, **sp.n.**, male. (**A**) antenna; (**B**) mesonotum and propodeum; (**C**) fore wing; (**D**) mesosoma and metasoma in ventral view.

#### 3.2.2. *Aphytis jinshanensis* Wang & Huang, **sp.n.** (Figure 7, Figure 8, Figure 9 and Figure 10)

Zoobank:urn:lsid:zoobank.org:act:2835381C-5E3A-4EE1-B11A-FEF79148E9C1

*Diagnosis. Aphytis jinshanensis*, **sp.n.** is considered to be related to, but not an actual member of, the *chrysomphali* group. This new species is closely related to *A. chrysomphali* (Mercet), but can be distinguished from it by: propodeum long, with a rather prominent median projection; crenulae clearly posteriorly elongate, 3 + 3 to 4 + 4 forming 1 set on the projection (*A.* *chrysomphali*: propodeum long, without a prominent median projection; crenulae not elongating backward, forming 2 sets). Thoracic sterna faintly dusky with a small black spot on each side of prothoracic sternum (*A. chrysomphali*: prothoracic sternum not marked a small black spot on each side).

*Description*. **Female.** Length 0.64–0.70 mm. **Colour.** Body yellow, mandibles black-brown; anterior margin of mid lobe of mesoscutum faintly dusky, two faintly dusky longitudinal stripe on the middle of scutellum, posterior margin of scutellum narrowly lined with black; thoracic sterna faintly dusky with a small black spot on each side of prothoracic sternum and a conspicuous longitudinal median black line on the stem of the mesosternal furca (“Y”). Legs and antennal scape concolorous with body; the base of pedicel, F2 and F3, the apex of club faintly dusky; Fore wing hyaline, faintly dusky at base and delta area. **Head.** Eyes finely setose. Mandible developed, with three sharp denticles. Antennal formula 1,1,3,1; scape slender, 4.69–5.80× as long as wide, longer than club; pedicel 1.40–1.63× as long as wide, longer than F3; F1 and F2 small; F3 0.92–1.09× as long as wide, somewhat rectangular, bearing 1 longitudinal sensillum; club 2.22–2.88× as long as wide, about 3× as long as F3, bearing 4–5 longitudinal sensilla. **Mesosoma.** Mid lobe of mesoscutum with 9–10 setae; each side lobe of mesoscutum with 2 setae; each axilla with 1 seta; scutellum with 4 setae, the placoid sensilla usually closer to the posterior than to the anterior pair of setae. Propodeum long, nearly as long as the scutellum, with a rather prominent median projection, reticulate broadly on the central area and weak on the sides; crenulae clearly posteriorly elongate, 3 + 3 to 4 + 4 forming 1 set on the projection, nonoverlapping. **Fore wing.** 2.87–3.00× as long as wide, marginal fringe not exceeding 0.33× width of disk; delta area with 33–38 setae in 4 or 5 rows; submarginal vein bearing 2 setae; marginal vein bearing 8 or 9 setae along anterior margin. **Leg.** Mid-tibial spur nearly as long as the corresponding basitarsus; tarsal formula 5-5-5. **Metasoma.** Petiole reticulate laterally, with weak transverse striations centrally. T1–T5 reticulate laterally, bearing 2 setae in each reticulate area, transverse striation across center. T5 and T6 with 2 setae centrally. T6 mostly reticulate. T7 triangular, bearing 6 setae in 1 row. Ovipositor located basally at T3, 1.35–1.43× as long as mid-tibia, third valvula short, 0.37× as long as mid-tibia.

**Figure 7:**
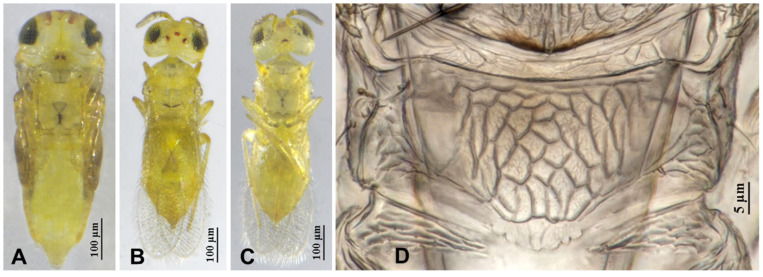
*Aphytis jinshanensis*, **sp.n.**, female. (**A**) pupa in ventral view; (**B**) adult in dorsal view; (**C**) adult in ventral view; (**D**) metanotum and propodeum.

**Figure 8:**
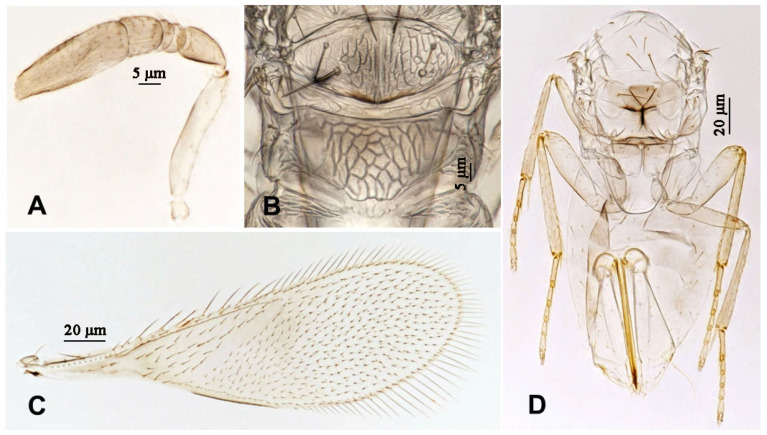
*Aphytis jinshanensis*, **sp.n.**, female. (**A**) antenna; (**B**) mesonotum and propodeum; (**C**) fore wing; (**D**) mesosoma and metasoma in ventral view.

**Male.** Length 0.64–0.70 mm. Essentially similar to the female in structure, chaetotaxy, sculpture and colour, differing mainly in the antennal proportions. Antenna shorter than that of the female, scape about 3.50× as long as wide, 1.10× as long as club; pedicel 1.39× as long as wide, 1.33× as long as F3; F1 and F2 small; F3 0.92× as long as wide, bearing 1 longitudinal sensillum; club 2.06× as long as wide, 2.92× as long as F3, bearing 2 longitudinal sensilla. Propodeum 0.90× as long as the scutellum, crenulae 2 + 2 to 2 + 3, as in the female. Male genitalia 0.76× as long as mid-tibia.

**Pupa.** The pupa of *A. jinshanensis*, **sp.n.** is yellow, with the wing pads and appendages slightly tinted with brown; thoracic sterna faintly dusky, with a small black spot on each side of prothoracic sternum and a longitudinal black line on the mesosternum.

**Figure 9:**
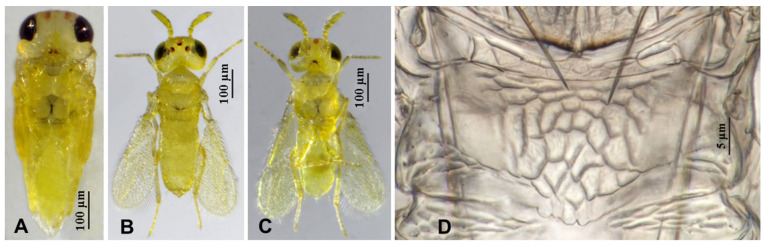
*Aphytis jinshanensis*, **sp.n.**, male. (**A**) pupa in ventral view; (**B**) adult in dorsal view; (**C**) adult in ventral view; (**D**) metanotum and propodeum.

**Figure 10:**
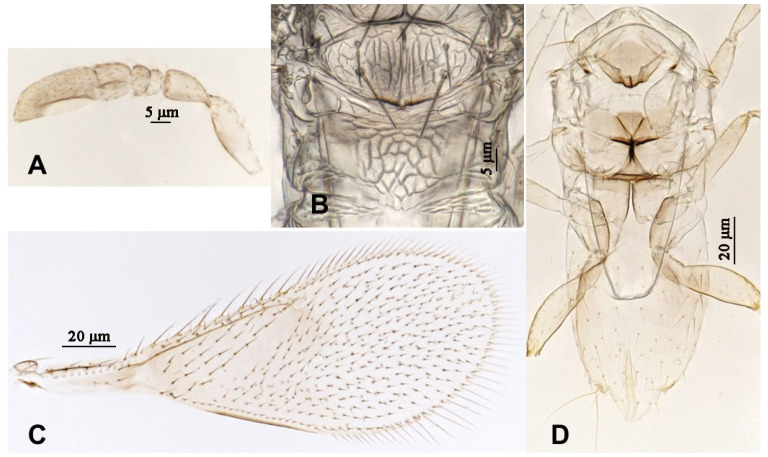
*Aphytis jinshanensis*, **sp.n.**, male. (**A**) antenna; (**B**) mesonotum and propodeum; (**C**) fore wing; (**D**) mesosoma and metasoma in ventral view.

*Host. Parlatoria ziziphi* (Lucas) on citrus.

*Distribution*. China (Fujian).

*Etymology*. The new species was named after the collection locality, Jinshan.

Material. **Holotype** ♀, China: Fujian, Fuzhou, Jinshan, FAFU, 22.xi.2023, **ex.** *Parlatoria ziziphi* (Lucas) on citrus. coll. Jingtao Xi (FAFU); **Paratypes** 3♀2♂, same data as holotype (FAFU).

Note. *A. jinshanensis*, **sp.n.** parasitizes the male of *P. ziziphi*.

#### 3.2.3. *Aphytis chrysomphali* (Mercet) [30,31] (Figure 11 and Figure 12)

*Aphelinus chrysomphali* Mercet, 1912, Boletin de la Real Sociedad Española de Historia Naturale, 12: 135–140. SPAIN (MNCN).

*Aphytis chrysomphali* (Mercet): New combination for *Aphelinus chrysomphali* Mercet by Timberlake, 1926, Proceeding of the Hawaiian Entomological Society, 6: 315; Rosen and DeBach, 1979, Species of *Aphytis* of the World, 593–598; Huang, 1994, Systematic Studies on Aphelinidae of China, 85–88.

**Figure 11:**
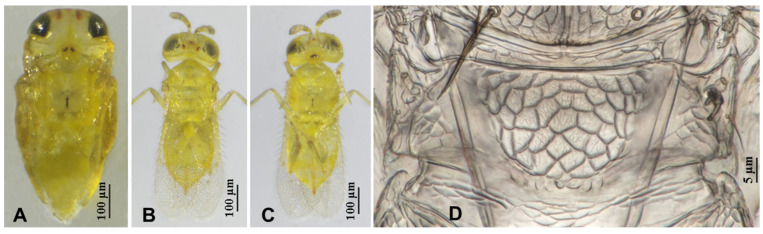
*Aphytis chrysomphali*, female. (**A**) pupa in ventral view; (**B**) adult in dorsal view; (**C**) adult in ventral view; (**D**) metanotum and propodeum.

**Figure 12:**
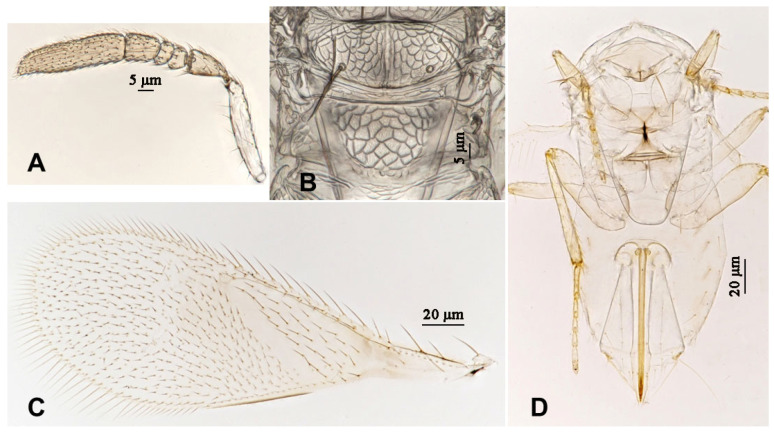
*Aphytis chrysomphali*, female. (**A**) antenna; (**B**) mesonotum and propodeum; (**C**) fore wing; (**D**) mesosoma and metasoma in ventral view.

*Host. Parlatoria ziziphi* (Lucas) on citrus, *Aonidiella aurantii* (Maskell), *A. citrina* (Coquillett), *Chrysomphalus dictyospermi* (Morgan), *Pseudaonidia trilobitiformis* (Green), *Melanaspis inopinata* (Leonardi), and this parasitoid is also recorded associated with some additional diaspidid scales [17,22].

*Distribution*. China, Japan, India, Europe, North America, South America, Pacific islands, Oceania, North Africa.

Material. 5♀, China: Fujian, Fuzhou, Jinshan, FAFU, 10.xi.2023, **ex.** *Parlatoria ziziphi* (Lucas) on citrus. coll. Jingtao Xi; 2♀, China: Hunan, Yongzhou, 10.xii.2024, **ex.** *P. ziziphi* on citrus. coll. Jingtao Xi (FAFU).

Note. *A. chrysomphali* parasitizes the male and early stage female of *P. ziziphi*. This *Aphytis* parasitoid is an uniparental species [17,22], and only females were collected during the study.

#### 3.2.4. *Aphytis* nr. *melinus* DeBach (Figure 13 and Figure 14)

*Host. Parlatoria ziziphi* (Lucas) on citrus.

*Distribution*. China (Fujian).

Material. 1♂, China: Fujian, Fuzhou, Jinshan, FAFU, 25.xi.2023, **ex.** *Parlatoria ziziphi* (Lucas) on citrus. coll. Jingtao Xi (FAFU).

**Figure 13:**
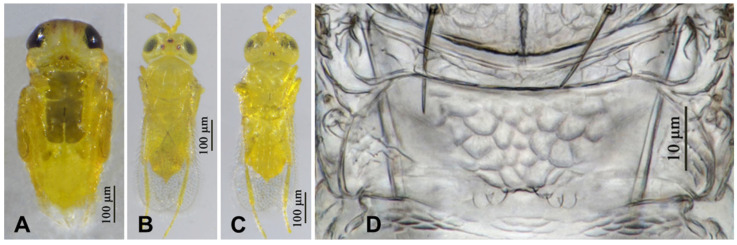
*Aphytis* nr. *melinus*, male. (**A**) pupa in ventral view; (**B**) adult in dorsal view; (**C**) adult in ventral view; (**D**) metanotum and propodeum.

**Figure 14:**
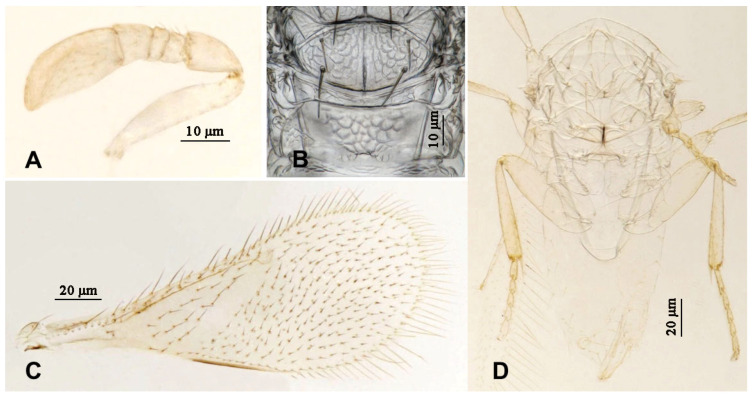
*Aphytis* nr. *melinus*, male. (**A**) antenna; (**B**) mesonotum and propodeum; (**C**) fore wing; (**D**) mesosoma and metasoma in ventral view.

Note. *A.* nr. *melinus* parasitizes the male of *P. ziziphi*. The pupa of *A.* nr. *melinus* is predominantly yellow, with the wing-pads and appendages slightly tinted with brown, and appears to be closely related to *A. melinus* DeBach [22] characterized by dark brown, well-defined pigmentation on the thoracic sterna, but differing from *A. melinus* mainly in a conspicuous longitudinal median black line on the stem of the mesosternal furca (“Y”).

#### 3.2.5. *Encarsia citrina* (Craw) [32,33] (Figure 15)

*Coccophagus citrinus* Craw, 1891, Bulletin of the California State Board of Horticulture, 57: 4. Syntypes 2♀, USA: Califonia, San Gabriel Valley, 1889, ex. *Aspidiotus citrinus*, (lost); Neotype ♀, designated by DeBach and Rose, 1981, Proceedings of the Entomological Society of Washington, 83: 671, same data as syntypes (USNM).

*Encarsia citrina* (Craw): New combination for *Coccophagus citrinus* Craw by Riley and Howard, 1891, Insect Life, 4: 167–168; Huang, 1994, Systematic Studies on Aphelinidae of China, 196–199; Huang and Polaszek, 1998, Journal of Natural History, 32: 1858–1860.

**Figure 15:**
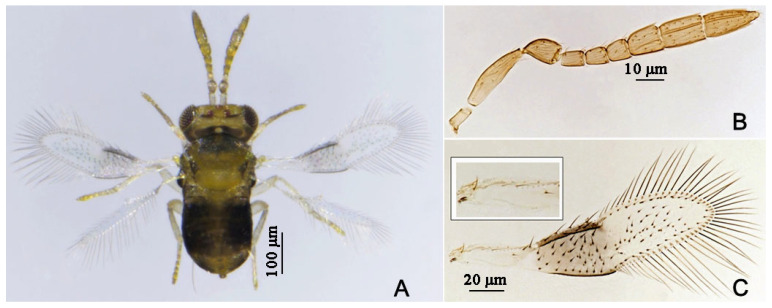
*Encarsia citrina*, female. (**A**) adult in dorsal view; (**B**) antenna; (**C**) fore wing and submarginal vein.

*Host. Parlatoria ziziphi* (Lucas) on citrus, *P. pergandii* Comstock, *Aonidiella aurantii* (Maskell), *A. citrina* (Coquillett), *Chrysomphalus aonidum* (Linnaeus), *Unaspis yanonensis* (Kuwana), *Hemiberlesia pitysophila* Takagi. This parasitoid is also recorded associated with a lot of diaspidid scales [17,34].

*Distribution*. China, Former USSR, Japan, India, Europe, North America, South America, Pacific islands, Australia.

Material. 4♀, China: Hunan, Yongzhou, 10.xii.2024, **ex.** *Parlatoria ziziphi* (Lucas) on citrus. coll. Jingtao Xi (FAFU).

Note. *Encarsia citrina* parasitizes the male and female of *Parlatoria ziziphi*.

### 3.3. Aphytis Species Having the Mid-Tibia Tipped with Black in the Male

The parasitoids of *Parlatoria ziziphi* are mainly *Aphytis* species (Table 1). *A. nigromaculata*, **sp.n.**, described here, is characterized by a black spot on the tip of mid-tibia in the male. So far there are only 5 species, out of total of about 100 species of *Aphytis*, having the mid-tibia tipped with black in the male. This work summarizes these 5 *Aphytis* species (Table 2) and provides an identification key to species as follows.

1. Propodeum 1.11–1.13x as long as scutellum………………………………………………………………………………………………………………..***A. cornuaspis***

-- Propodeum 0.75–0.90x as long as scutellum………………………………………………………………………………………………………………………………2

2. Fore wing with unequal setae along the marginal vein; antennal scape with specialized sensilla in the male………………………………..***A. salvadorensis***

-- Without characteristics as above……………………………………………………………………………………………………………………………………………3

3. Mandibles bidentate…………………………………………………………………………………………………………………………………………..***A. erythraeus***

-- Mandibles tridentate or with 2 denticles and a dorsal truncation……………………………………………………………………………………………………..4

4. Mid-tibia faintly tipped with brownish in the female; mid-basitarsus black on basal half in the male; antennal F3 in the male obliquely truncate from the dorsal to the ventral, ventral aspect considerably longer than the dorsal; the pupa entirely yellow…………………………………………………..***A. mazalae***

-- Mid-tibia yellow in the female; mid-basitarsus yellow in the male; antennal F3 in the male obliquely truncate from both the dorsal and ventral to the central, forming a point; the pupa with head and thoracic sterna blown to dark brown...……………………………………………….***A. nigromaculata*, sp.n.**

### 3.4. Phylogenetic Analysis

**Figure 16:**
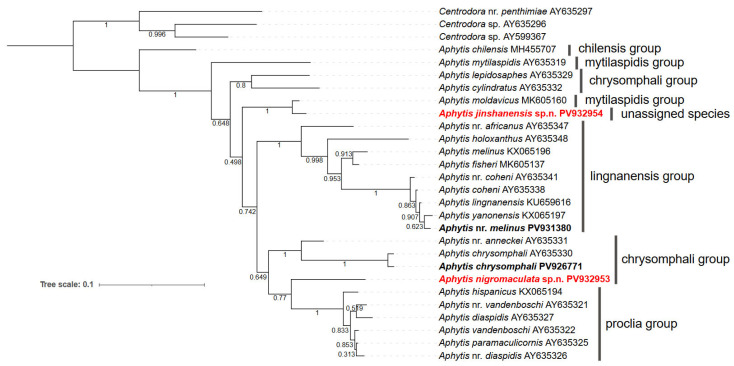
Bayesian tree based on 28SrDNA-D2 sequences. Posterior probability values are shown for nodes.

In the Bayesian analysis (Figure 16), three *Centrodora* species (Aphelinidae) are the outgroup and the *Aphytis* species cluster on a large branch, in which the species of *lingnanensis* and *proclia* groups each cluster on a branch (1.00 posterior probability). The species of the *chrysomphali* group irregularly form 2 distinct branches. This group is probably polyphyletic in origin. *Aphytis lepidosaphes* and *Aphytis cylindratus* are distantly related to the other three species of the *chrysomphali* group. In morphology, *Aphytis lepidosaphes* and *Aphytis cylindratus* belong to the *chrysomphali* group, but their characteristics are closely related to the other groups. *Aphytis nigromaculata*, **sp.n.** is placed in a branch (0.77 posterior probability) with the species of *proclia* group, but it indeed belongs to the *chrysomphali* group. While *Aphytis jinshanensis*, **sp.n.** is independent from the branch of the *chrysomphali*, *lingnanensis* and *proclia* groups, and clusters on the branch with *Aphytis moldavicus.*

## 4. Discussion

*Aphytis* species are the dominant parasitoids of *Parlatoria ziziphi*. However, it is difficult to identify the species, particularly those in the *lingnanensis* group, based on adult morphological characters. Wang et al. (2021) [35] presented four characteristic pigmentation patterns of *Aphytis* pupae: entirely yellow, partly dark brown, entirely or predominantly black, and partly black pupae, and pointed out that the pupal pigmentation characters are considered as an important supplementary diagnostic character for distinguishing species of *Aphytis*, especially closely-related species, as well as the adult morphological characters. In this work, *A.* nr. *melinus* belongs to the *lingnanensis* group, and appears to be closely related to *A. melinus* DeBach characterized by the pupa with dark brown, well-defined pigmentation on the thoracic sterna, but differing from *A. melinus* mainly by having a conspicuous longitudinal median black line on the stem of the mesosternal furca (“Y”). Also, *A.* nr. *melinus* has a significant genetic distance from *A. melinus* in the Bayesian analysis (Figure 16). As only one male individual of *A*. nr. *melinus* was collected, further identification of this species was not possible.

Molecular DNA analysis supports the status of *Aphytis jinshanensis,* **sp.n.** as an unassigned species, and it is closely related to *A. moldavicus*. In the Bayesian analysis (Figure 16), *A. nigromaculata*, **sp.n.** is placed in a branch with the *proclia* group, but they are significantly genetically separated. Actually, *A. nigromaculata*, **sp.n.** belongs to the *chrysomphali* group by characters and resembles *A. mazalae* DeBach & Rosen in the *chrysomphali* group.

In Table 1, the 2 species of Encyrtidae on *P. ziziphi* were recorded by Coll and Abd-Rabou (1998) [5], Yang (2004) [19] and Abd-Rabou and Badary (2012) [20], respectively. In our survey, however, all the parasitoids of *P. ziziphi* are Aphelinidae, with no Encytidae. So there is also a need for further and extensive investigation of parasitoids associated with *P. ziziphi*.

## Figures and Tables

**Table 1:** Known primary and secondary parasitoids of *Parlatoria ziziphi* (Lucas).

Parasitoids (Family/Species)	Distribution	References
Aphelinidae		
*Aphytis chrysomphali*	China	[12], Note in this paper
*A. hispanicus*	Egypt	[13]
*A. lingnanensis*	Egypt	[14]
*A. melinus*	Egypt	[11]
*Encarsia citrina*	China, Egypt	[5,10,12,14,15,16,17]
*E. lounsburyi*	Iran	[18]
** *Prospaltella inquirenda*	Egypt	[13]
* *Marietta leopardina*	Egypt	[5]
Encyrtidae		
*Comperiella bifasciata*	China	[19]
** *Habrolepis aspidioti*	Egypt	[5]
*H. diaspidi*	Egypt	[20]

Note. * hyperparasitoid. ** *Prospaltella inquirenda* is now *Encarsia inquirenda* [17]; *Habrolepis aspidioti* is a synonym of *Habrolepis diaspidi* [21].

**Table 2:** *Aphytis* species having the mid-tibia tipped with black in the male.

Species	Host	Distribution	References
*Aphytis nigromaculata* **sp.n.**	*Parlatoria ziziphi* on citrus	China (Fujian)	Described in this paper
*A. cornuaspis*	*Lepidosaphes gloverii* on citrus	China (Fujian)	[17]
*A. erythraeus*	*Aspidiotus elaeidis* on olive	Africa	[22]
*A. mazalae*	*Aulacaspis murrayae* on *Murraya funiculara*, *Aonidiella citrina* on *Citrus sinensi*, *Pinnaspis strachani* on *Ficus palmata*	China (Taiwan), Japan, Pakistan	[22]
*A. salvadorensis*	an undetermined scale insect	El Salvador	[22]

**Table 3:** Primers and cycling conditions.

Primer	Sequence	Cycling Conditions			
D2-3551F	5′-CGTGTTGCTTGATAGTGCAGC-3′	Denaturation	Annealing	Extension	Cycles
D2-4068R	5′-TTGGTCCGTGTTTCAAGACGGG-3	94 °C (30 s)	58 °C (30 s)	72 °C (60 s)	35

**Table 4:** The species and GenBank accession numbers used in phylogenetic analysis.

Genus/Species	GenBank Accession	Source	Genus/Species	GenBank Accession	Source
Genus *Aphytis*			*A. lepidosaphes*	AY635329	GenBank
*A.* nr. *anneckei*	AY635331	GenBank	*A. lingnanensis*	KU659616	GenBank
*A.* nr. *africanus*	AY635347	GenBank	*A. melinus*	KX065196	GenBank
***A. chrysomphali***	PV926771	This paper	***A.*** **nr. *melinus***	PV931380	This paper
*A. chrysomphali*	AY635330	GenBank	*A. moldavicus*	MK605160	GenBank
*A. chilensis*	MH455707	GenBank	*A. mytilaspidis*	AY635319	GenBank
*A. cylindratus*	AY635332	GenBank	***A. nigromaculata,*** **sp.n.**	PV932953	This paper
*A. coheni*	AY635338	GenBank	*A. paramaculicornis*	AY635325	GenBank
*A.* nr. *coheni*	AY635341	GenBank	*A. vandenboschi*	AY635322	GenBank
*A. diaspidis*	AY635327	GenBank	*A.* nr. *vandenboschi*	AY635321	GenBank
*A.* nr. *diaspidis*	AY635326	GenBank	*A. yanonensis*	KX065197	GenBank
*A. fisheri*	MK605137	GenBank	Genus *Centrodora*		
*A. hispanicus*	KX065194	GenBank	*Centrodora* sp.	AY635296	GenBank
*A. holoxanthus*	AY635348	GenBank	*C.* nr. *penthimiae*	AY635297	GenBank
***A. jinshanensis*** **, sp.n.**	PV932954	This paper	*Centrodora* sp.	AY599367	GenBank

## Data Availability

The original contributions presented in this study are included in the article. Further inquiries can be directed to the corresponding author.
